# A Simulation Method for Life Cycling Aging of Asphalt Binders Under Temperature Cycling Effects

**DOI:** 10.3390/ma18071542

**Published:** 2025-03-28

**Authors:** Yiwen Tong, Yamin Liu, Xuhui Han, Yuze Tan

**Affiliations:** 1 Sanming Highway Development Centre, Sanming 365000, China; tyw619@163.com; 2 Key Laboratory for Special Area Highway Engineering of Ministry of Education, Chang’an University, Xi’an 710064, China; xuhuihan@chd.edu.cn (X.H.); tanyuze2024@163.com (Y.T.)

**Keywords:** asphalt binder, aging asphalt, laboratory aging tests, thermal oxidation, life cycling aging, dynamic shear rheology, temperature aging test, test parameters

## Abstract

Laboratory simulation is an effective method to obtain asphalt at different aging stages, like short-term aging through the Rotated Thin Film Oven Test (RTFOT) and long-term aging through a Pressure Aging Vessel (PAV). However, these methods have certain limitations: RTFOT produces small amounts of asphalt and is hard to clean, and PAV is costly. This paper presents a simple and economical way to obtain a large amount of aged asphalt with a single device called the Temperature-Cycle Aging Oven Test (TCAOT). Some key experimental parameters were investigated, including experimental conditions such as whether only thermal aging or fusion ultraviolet (UV) aging is required, the test temperature, sample quantity, sample placement, and termination criteria, etc. The rheological properties of aged asphalt from the new method were compared with traditional asphalt. The results show that the complex shear modulus after thermal aging is similar to that with UV radiation. Due to the difficulties, 125 g of asphalt is advised. For short-term aging, TCAOT at 163 °C for 120 min is like RTFOT. For long-term aging, TCAOT at 95 °C for 72 h is like PAV. With more aging, TCAOT at 95 °C for 120 h is equal to 15 years of real asphalt pavement aging. Based on TCAOT’s excellent test performance, transportation and road construction departments can consider adopting this method to improve operational efficiency and save costs to increase revenue.

## 1. Introduction

Over 90% of high-grade road surfaces are made of asphalt pavement, which usually has a 10- to 15-year service life. But during its service life, aging presents a serious problem. Minmin Xiao et al. [1] used molecular dynamic simulation methods to study the aging process of asphalt under ultraviolet irradiation and found that aging asphalt modifies the binder structure, causing it to lose its properties and cause distress in the pavement, which, in turn, shortens the pavement’s life. However, the interaction between multiple factors in the actual service environment, such as thermal oxygen aging and water aging, has not been considered, which limits the depth of this mechanism’s explanation.

The aging of asphalt binders is a lengthy process, typically ranging from several years to several decades. In order to achieve aging effects equivalent to actual road conditions in a shorter period of time, the laboratory simulation of asphalt aging behavior is currently the main experimental method.

Thermal oxidation aging (temperature) and photo-oxidation aging (ultraviolet, hereinafter referred to as UV) are two important factors causing asphalt aging. Most existing aging simulation methods mainly focus on thermal oxidation aging, and there is still significant controversy regarding whether UV aging should be considered.

Shihui Guo et al. [2] attempted to establish the equivalence between UV aging and natural aging. The results showed that the complex modulus and zero shear viscosity of the base asphalt binder gradually increased while the phase angle decreased with prolonged UV aging time. As such, 16–18 days of UV aging are equivalent to 18 months of natural aging. But in the aging test, the thickness of the asphalt samples is 3.2 mm, and for the actual pavement, this may be different, which could affect the aging rate further. Wenbo Zeng et al. [3] used UTS tests and the peeling method to investigate the UV aging depth of asphalts. However, these studies only considered the effect of UV aging on the asphalt binder, which is not consistent with the actual situation. The analysis concluded that the direct effect of UV aging is limited in scope; the asphalt layer within 4.5 μm is subjected to direct aging, which then gradually diffuses to the underlayer (under 4.5 μm) of asphalt. Depending on the stage of aging, the asphalt binder can be classified into short-term aging and long-term aging. Many studies have investigated these aging processes. However, UV aging possesses few aging standards, and these studies only consider the effects of UV aging on asphalt binders, which does not correspond to the actual situation. According to the aging stage, the aging of asphalt cement can be divided into short-term aging and long-term aging. Many studies have investigated these aging processes.

Hao Yu et al. [4] adopted the Thin Film Oven Test (TFOT) to simulate short-term aging. A total of 50 g of asphalt was used to create a 3.0 mm thick asphalt film in a 140 mm diameter dish for short-term aging. The conclusion was that after 24 h of TFOT aging, the low-temperature performance of micro-SBS-modified asphalt significantly deteriorated, and the high-SBS content asphalt performed better. The limitation is that excessively high temperatures and a long time may lead to the thermal decomposition of asphalt rather than real oxidative aging, which weakens the engineering reference value of the conclusion. Hveem et al. [5] proposed RTFOT as a means of simulating short-term asphalt aging, improving TFOT. The asphalt sample was reduced to 35 g, and the asphalt was exposed to more oxygen by constantly moving within the aging bottle in place of the previous dish. The aging time was reduced to 85 min as a result. However, the amount of aged asphalt produced by this test was relatively small, and the aging bottles were difficult to clean. Some studies [6,7,8] have found that there are some problems with using RTFOT to simulate the short-term aging of modified asphalt, such as the high-temperature gradation of modified asphalt decreasing after short-term aging, which is clearly inconsistent with the test results of the matrix asphalt.

Additionally, Siyue Zhu et al. [9] used PAV to simulate long-term aging. This method could age 500 g of samples at a time, and it was widely used. In this paper, the evolution of the long-term rheological properties of high-viscosity asphalt was effectively revealed through PAV aging. However, due to the uniformity of laboratory aging conditions and the limitations of the test indexes, it may not be able to fully reflect the aging behavior in the actual complex environment. Qin Q et al. [10] found through the comparative analysis of laboratory-simulated aging tests and field aging results that PAV aging levels corresponded only to the aging level of field pavements after 8 years of use. Fang Liu et al. [11] found that the high-pressure conditions of PAV may cause some errors in the results. Therefore, some researchers have attempted to find more convenient methods to simulate asphalt binder aging. Li Peilong et al. [12] prolonged the aging time of RTFOT to simulate long-term aging and found that the aging asphalt binder for 270 min in RTFOT could achieve the same aging effect as PAV. In their latest study, Jianbing Ma et al. [13] compared the effects of different aging methods (RTFOT, PAV, UV) and different UV aging times (50 to 200 h) on WMA. The results showed that UV aging had the greatest effect on the adhesion and functional group index of WMA, followed by PAV aging, and RTFOT aging had the worst effect. Areas for improvement include the fact that the synergistic mechanism of oxygen and UV is not deeply explored at the molecular level, and the contribution rate is only quantified by statistical models.

Recently, Michael J Farrar et al. [14] proposed a new test method for the oxidative aging of thin films, SAT, as an alternative to the traditional RTFO and PAV. SAT combines 4 mm parallel plate DSR technology to assess the rheological properties of bitumen at both low and high temperatures. Furthermore, Michael J. Farrar et al. [15] used the SAT method in conjunction with PAV to reduce the aging time from 20 h to 8 h. The disadvantage to this is that the ratio of asphalt to water is estimated in advance to ensure the film’s thickness, which can introduce errors. Zhuang Zhang et al. [16] used the SAT method to evaluate the effect of different aging times on the properties of SBS-modified asphalt and compared it with the traditional RTFOT and PAV methods. The results show that the SAT method has a strong correlation with RTFOT and PAV in simulating the aging effect. In fact, only two SBS contents (3% and 4%) were tested without covering a wider range of different types of modifiers (such as other polymers or recycled materials). K Primerano et al. [17] used PAV and VBA to age bitumen and compared this with field-aged samples. When analyzed by FTIR, DSR, SARA fractionation, and fluorescence spectroscopy, VBA was found to approximate field aging better in some respects. SAT involves pouring asphalt into a mold to form 300 μm of film thickness, and this is used to subsequently age the asphalt sample in an ordinary oven at 150 °C for 50 min to achieve short-term aging. Long-term aging is accomplished in two ways: either by placing the short-term aged sample in an ordinary oven at 100 °C for 40 h or by subjecting the aged asphalt to 100 °C of PAV for 8 h. However, whether the aging mechanism of asphalt binders under this light source aligns with natural aging in the environment requires further investigation.

From the studies discussed above, although RTFOT and PAV have been widely used, there are still certain limitations for these aging methods: the amount of asphalt obtained from RTFOT is very small, and the bottles are difficult to clean, while PAV is expensive, especially for developing countries. In addition, the coupling effect of multiple aging factors is difficult to achieve with a single device. Therefore, an economical and convenient device or method is highly needed to study the life-cycling aging of asphalt binders. The overall objective of this study is to introduce how such a device works.

The main findings of this study are as follows: (1). Compared to other methods, the Temperature-Cycle Aging Oven Test has outstanding advantages, such as accuracy, efficiency, and low costs. (2). With the Temperature-Cycle Aging Oven Test, asphalt can achieve conditions of short-term aging, long-term aging, and life-cycling aging simultaneously. (3). The actual aging level of asphalt pavements over 15 years in the field corresponds to the aging effect of asphalt achieved under TCAOT conditions of 95 °C for 120 h in the laboratory.

This paper introduces a simple and economical experimental method to obtain a large quantity of aged asphalt, achieved through a single device called the Temperature-Cycle Aging Oven Test (TCAOT). The following chapters are arranged as follows: First, the materials and methods used in the test are introduced. The structure and operation procedure of TCAOT are introduced in detail. Next, some key experimental parameters are investigated, including experimental conditions and whether only thermal aging is needed or fusion ultraviolet (UV) aging is required, the test temperature, sample quantity, sample placement, termination criteria, etc. Similarly, the rheological properties of aged asphalt obtained from the new method are compared and analyzed with those obtained from traditional methods. Finally, the necessity of life-cycling aging is analyzed.

## 2. Materials and Methods

### 2.1. Materials

Three types of asphalt were included in this study. Sample types and corresponding regular performance-based properties are listed in Table 1. The data in Table 1 were obtained by referring to the trials in the Standard Test Methods of Bitumen and Bituminous Mixtures for Highway Engineering (JTG E20—2011) [18].

### 2.2. Methods

#### 2.2.1. Aging Simulation Device and Operating Procedures

ⅰ Equipment Composition.

**Figure 1:**
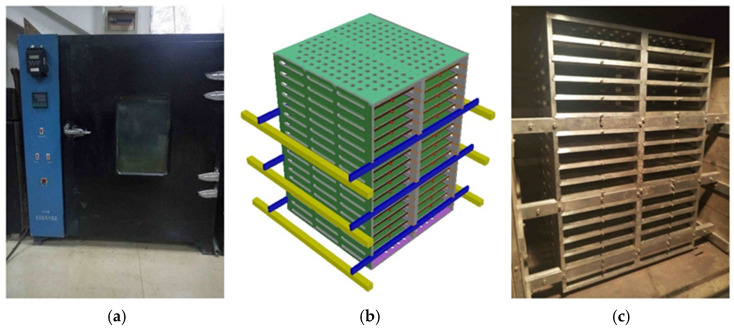
Equipment composition: (**a**) exterior appearance; (**b**) 3D view of the aging stent; and (**c**) physical picture of the aging stent.

The TCAOT aging simulation device primarily consists of two components: the oven and the aging support rack, as depicted in Figure 1.

(1) Oven.

The oven has dimensions of 800 mm × 800 mm × 800 mm and consists of two primary components: the heating system and the circulation system. The heating system employs an air bath heating method with an automatic temperature regulator capable of controlling the oven environment within the range of 0 to 200 °C. The circulation system operates via blowers on both sides of the oven, ensuring the continuous circulation of internal hot air to maintain uniformity in the oven’s temperature environment.

(2) Aging Support Rack.

**Figure 2:**
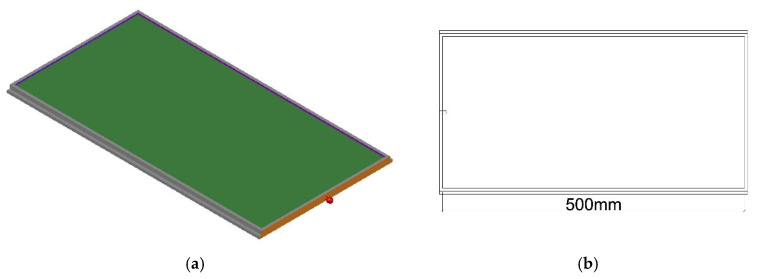
Aging tray construction: (**a**) 3D view; (**b**) plan view.

The aging support rack is fashioned from three whole pieces of 12 mm aluminum plates, segmented into aging trays, upper cover plates, lower cover plates, side panels, intermediate partitions, and support frames. As depicted in Figure 2b, the aging support rack is suspended inside the oven, and its overall size should be smaller than the internal dimensions of the oven, ensuring a specific gap between the aging support rack and its surroundings, facilitating internal hot air circulation. The flatness of the aging support rack is maintained by regulating the six transverse support rods on either side of the aging rack. The detailed construction of each component is as follows:

① Aging Trays.

The structure of the aging trays, as illustrated in Figure 2, has overall dimensions of 510 mm (length) × 270 mm (width) × 12 mm (height), including a 500 mm (length) × 250 mm (width) × 3 mm (thickness) groove for holding onto the asphalt. In order to store conveniently, the aging trays are fixed to the aging support rack in a slide rail manner (similar to drawers), with protrusions on both sides serving as rails for support and fixation. The aging support rack accommodates a total of 32 aging trays. Both the trays and rack are precision-machined using high-precision CNC machines (machining accuracy of 0.02 to 0.05 mm), ensuring the trays’ flatness by controlling the aging support rack. Each tray holds 125 g of the asphalt binder, enabling aging of up to 4 kg for the asphalt binder in a single test.

② Upper and Lower Cover Plates.

**Figure 3:**
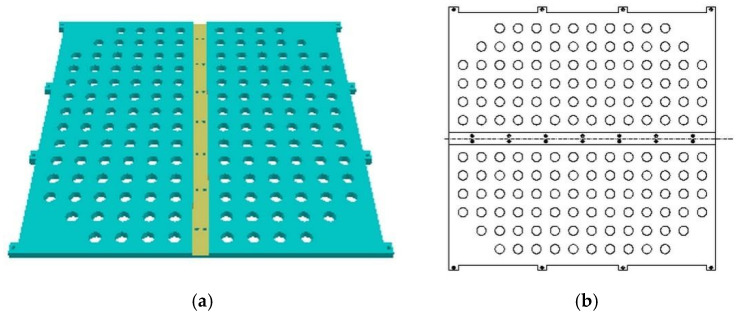
Construction of upper and lower cover plates: (**a**) 3D view; (**b**) plan view.

The upper and lower cover plates share identical dimensions and structures, as depicted in Figure 3. The overall dimensions of the cover plates measure 580 mm (length) × 549 mm (width) × 12 mm (height). Protrusions around the perimeter facilitate the assembly of the upper and lower cover plates with side panels, secured using rivets. The groove in the middle has a depth of 5 mm and is used for splicing the upper and lower cover plates and the middle partition board. It is also fixed with rivets. To ensure uniform aging by facilitating the circulation of hot air within the oven, holes with a diameter of 20 mm and spaced 40 mm apart are drilled on the cover plates.

③ Side Panels.

**Figure 4:**
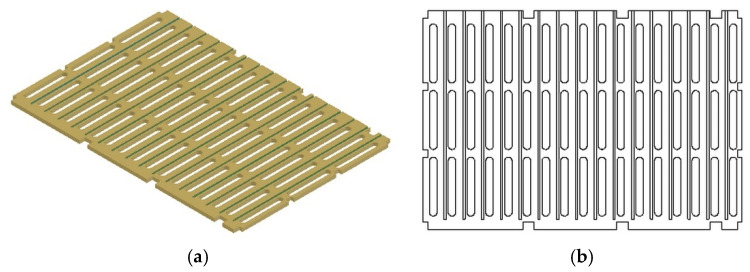
Construction of side panels: (**a**) 3D view; (**b**) plan view.

As shown in Figure 4, the construction of the side panels measures 850 mm (length) × 580 mm (width) × 12 mm (height). The grooves around the perimeter serve as junction points between the side panels and the supporting framework, while the central hollow design promotes air circulation. The grooves depicted in Figure 4a act as rails (6 mm width, 6 mm depth) for the placement of aging tray tracks, spaced at 50 mm intervals.

④ Intermediate Partition.

**Figure 5:**
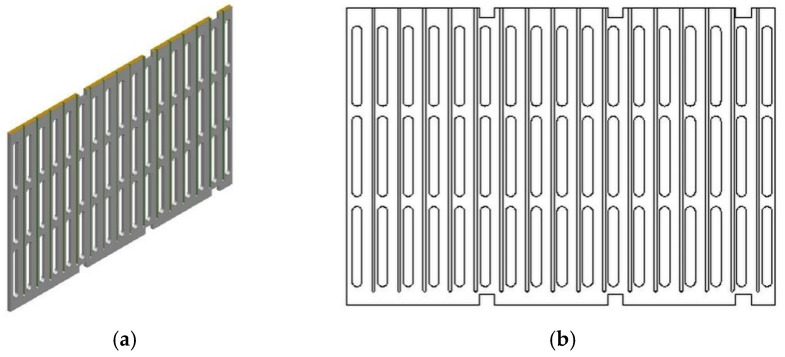
Intermediate partition construction: (**a**) 3D view; (**b**) plan view.

The overall structure of the intermediate partition is similar to the side panels, as seen in Figure 5. However, the length of the intermediate partition is slightly smaller, measuring 836 mm without grooves at the top and bottom. Considering the need for guide rails on both sides of the partition and its relatively thin thickness of 12 mm, the intermediate partition is assembled by joining two separate panels.

ⅱ. Operational Procedure.

The operational procedure for the TCAOT aging simulation device is outlined as follows:

(1) Preparatory Work.

① Ensure the aging trays are cleaned beforehand using solvents such as gasoline or trichloroethylene and dried in an oven at 105 °C (±0.5 °C).

② Ensure the oven is level and activate the heating and blower switches, allowing the oven to reach the aging test temperature within 10 min, reaching 163 °C (±0.5 °C), and preheat the oven for a minimum of 16 h to ensure uniform temperature distribution.

③ Following the sampling method per test protocol [18], pour 125 g (±0.5 g) of asphalt into each aging tray.

(2) Test Steps.

① Short-Term Aging.

Place the aging tray containing the asphalt sample on the aging support rack within 5 min. The oven temperature should rise to the test temperature of 163 °C (±0.5 °C) within 15 min, and the sample should be heated for a minimum of 105 min at this temperature, totaling 120 min of heating time.

② Long-Term Aging.

After completing short-term aging, adjust the oven temperature to 95 °C (±0.5 °C) for long-term aging, and ensure the oven reaches the set temperature within 20 min.

③ Sampling of Aged Asphalt Binder.

**Figure 6:**
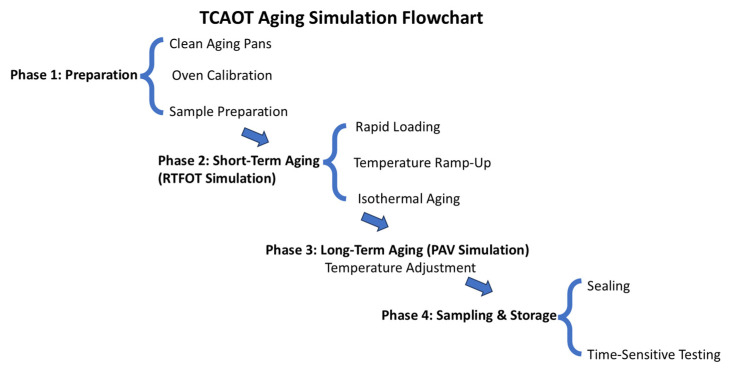
TCAOT aging simulation flowchart.

Place the aged asphalt binder into a sealed aluminum box, as depicted in Figure 6. All performance tests should be completed within 72 h.

(3) Precautions.

To avoid test errors impacting the analysis results, it is recommended to adhere to the following steps during the experimental procedures:

① To prevent sampling discrepancies from affecting test results, it is advisable to evenly divide one type of asphalt sample and strive for consistent heating times for each test sampling.

② Utilize the same aging tray for each experiment; place it in the same position on the support rack during aging to avoid data variations due to differences in tray levels.

③ To maintain the consistent circulation of hot air within the oven and avoid the influence of different tray quantities, ensure the number of aging trays inside the oven remains as consistent as possible for each test.

#### 2.2.2. DSR Measurements

Dynamic Shear Rheometer (DSR) temperature scanning tests were used to determine the dynamic shear modulus and phase angle of the asphalt. The measured values of the dynamic shear modulus of asphalt ranged from 0.1 to 10 MPa. The fabricated specimens were tested immediately after immersion in a prescribed constant-temperature water bath. In the case of setting multiple test temperatures, it is desirable to control the whole test process within 4 h. The working principle of the DSR measurements is that the asphalt sample is placed between the oscillating plate and the stationary plate, which is subjected to cyclic movement by the oscillating plate.

The DSR measurements were conducted to investigate the variations and patterns in the rheological properties of asphalt under different aging methods and test parameters.

## 3. Results and Discussion

### 3.1. Test Conditions

Thermal oxidation (temperature) and photo-oxidation (UV radiation) are two crucial factors causing asphalt aging. To delineate the varying degrees of influence between thermal and photo-oxidation on asphalt binder properties, this study conducted Dynamic Shear Rheometer (DSR) tests under four aging conditions on ZH-70# asphalt: initial, thermal aging, photo-oxidation aging, and thermal + photo-oxidation coupled aging, over a period of 30 days at a thermal aging temperature of 60 °C. Following ASTM G154 [19], UVA-340 was chosen as the laboratory-simulated light source for photo-oxidation to match the solar radiation intensity in nature. Hence, with the aim of approximating real aging effects, this study utilized UVA-340 as the light source at a test temperature of 60 °C.

**Figure 7:**
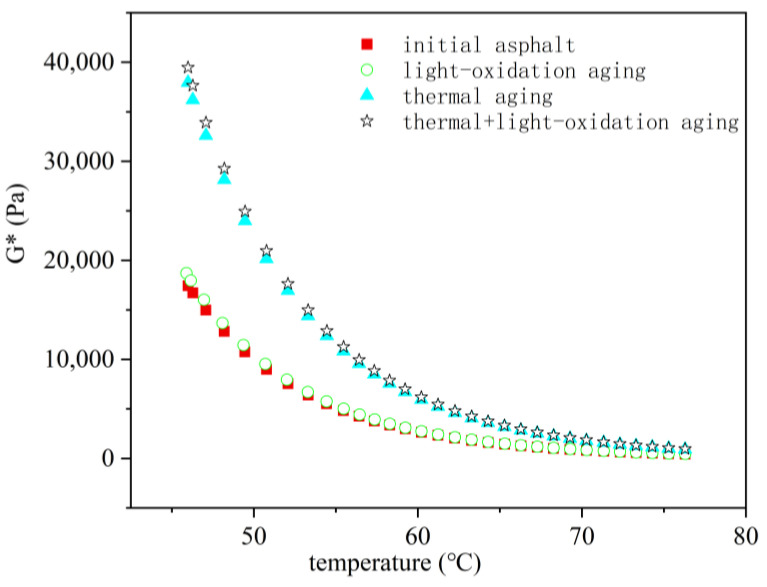
Variation in the complex shear modulus G* of ZH-70# asphalt.

Figure 7 illustrates the variation curve of complex shear modulus (G*) for ZH-70# asphalt under different test conditions. It can be seen that when UVA-340 was used as the light source, the difference in G* between the asphalt binder subjected to photo-oxidation aging and unaged asphalt was minimal. However, the G* of the thermally aged asphalt binder was akin to that of asphalt aged under temperature + UV radiation, indicating that thermal oxidation has a more pronounced effect on asphalt binder properties compared to UV radiation. Moreover, under coupled conditions, thermal oxidation takes precedence. Some researchers [20,21,22] argue that the radiation intensity of UVA-340 is insufficient to effectively accelerate aging, opting to substitute it with mercury lamps. Although mercury lamps accelerate aging, it remains uncertain whether the aging mechanism of the asphalt binder under this light source aligns with natural environmental aging mechanisms. UV aging has a significant effect on asphalt film with a thickness of 3 mm, while the effect on thicker film is slight. In addition, it is said that UV aging affects only the top layer of the asphalt binder [23,24,25]. Under thermal oxidation aging conditions, air and heat can easily circulate over the surface of the asphalt [26,27]. This allows the asphalt to be aged uniformly. Considering these findings, this study chose thermal oxidation as the aging method, specifically temperature-based aging.

### 3.2. Test Temperature

The aging simulation primarily employs thermal oxidation. Hence, the test temperature becomes a critical controlling condition influencing the effectiveness of asphalt binder aging. The test temperature should be chosen to both accelerate the aging process and to ensure that the laboratory aging process is as close as possible to the real situation.

Firstly, in order to determine the short-term aging test temperature, considering the specific state of asphalt binders during storage, transport, and paving processes, the short-term aging temperature of the TCAOT simulation equipment was set at 163 °C (±0.5 °C), which is consistent with the RTFOT temperature.

Secondly, to determine the long-term aging test temperature, an extensive literature review was conducted. The asphalt pavement surface temperature is closely related to environmental temperature and geographical location. During peak summer temperatures, most asphalt pavement surface temperatures range around 60 °C, with some areas even reaching 70 °C [28,29]. In order to accelerate aging, the long-term aging test temperature should not be lower than 70 °C while also not exceeding 100 °C [30]. Temperatures surpassing 100 °C led to the increased volatilization of lighter components or polymer degradation in asphalt binders, which contradicts real-world scenarios where such phenomena do not occur during long-term aging processes.

**Figure 8:**
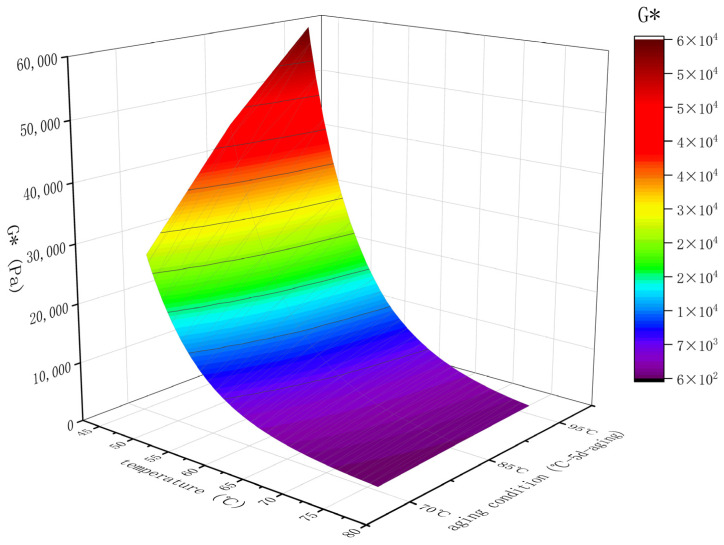
Results of DSR temperature scanning test on ZH-70# asphalt at different temperatures.

Thus, to ascertain the long-term aging test temperature, this study conducted aging for a period of time (5 days) at different temperatures (70 °C, 85 °C, and 95 °C) for ZH-70# asphalt. DSR temperature scanning tests were then conducted on the aged asphalt binders to analyze differences in their rheological properties, as depicted in Figure 8.

The data in Figure 8 concluded that with increasing temperature, there was a significant change in asphalt G* after aging. Assuming that G* corresponds to 46 °C as an evaluation parameter, the G* at 95 °C was 1.3 times and 2.2 times higher compared to 85 °C and 70 °C. This indicates that asphalt aging is more severe at higher test temperatures for the same aging time, implying that higher temperatures require less time to achieve equivalent aging effects. This conclusion aligns with Elwardany’s study [31], where microscale evaluation indicators are used to analyze asphalt binder aging performance.

Therefore, this study determined the long-term aging temperature of the TCAOT simulation equipment to be 95 °C (±0.5 °C).

### 3.3. Sample Quantity

Determining the sample quantity requires considering both the aging rate and the recoverable amount of asphalt after aging. Oversized or undersized sample quantities could affect the final aging outcomes or subsequent experiments. Most asphalt aging simulation methods use the thickness of asphalt film (mm) as a benchmark.

While the thickness of the film does not affect asphalt oxidation reaction mechanisms, reducing the thickness of the film can accelerate the aging reaction rate. Based on the PAV test results for asphalt specimen size and aging tray dimensions, the estimated thickness of the asphalt film is 3.2 mm.

In this study, using DSR temperature scanning tests, asphalt with different film thicknesses (0.5 mm, 1 mm, and 2 mm) were analyzed under the same aging level (temperature: 95 °C, time: 5 days).

**Figure 9:**
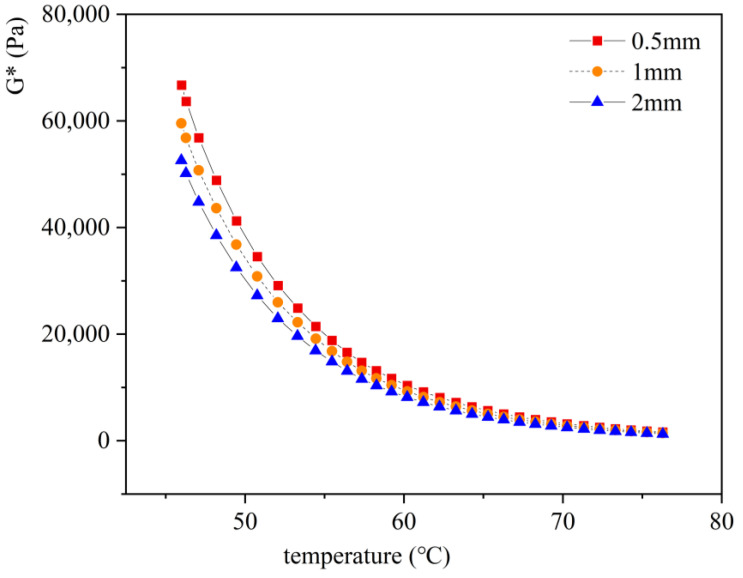
Results of DSR temperature scanning test on ZH-70# asphalt under different thicknesses and asphalt film conditions.

Figure 9 presents the results of the DSR temperature scanning test for ZH-70# asphalt under different thicknesses and asphalt film conditions. The observations were as follows:

(1) Thinner asphalt films exhibited higher G*, indicating more severe aging. Assuming G* at 46 °C as the evaluation index, G* under the asphalt film 0.5 mm thick is 1.12 and 1.27 times the G* of the asphalt film that is 1 mm and 2 mm thick.

(2) For a 0.5 mm thick asphalt film, the recovered quantity after aging was approximately 50 g, which was insufficient to complete subsequent tests like penetration and ductility. On the other hand, a 2 mm thick asphalt film resulted in excessive asphalt quantity, leading to flow and overflow during the experiment, not only affecting aging effectiveness but also contaminating the aging frame, making it difficult to clean.

Hence, a 1 mm thick asphalt film was determined at the optimal thickness. Based on aging tray dimensions and the thickness of the asphalt film, the calculated quantity of asphalt used in the experiment was 125 g.

### 3.4. Sample Placement

To enhance experimental efficiency, 32 trays were created for the aging tests in the simulation equipment. However, varying tray placements might induce differences in asphalt binder aging effects during the experiments.

**Figure 10:**
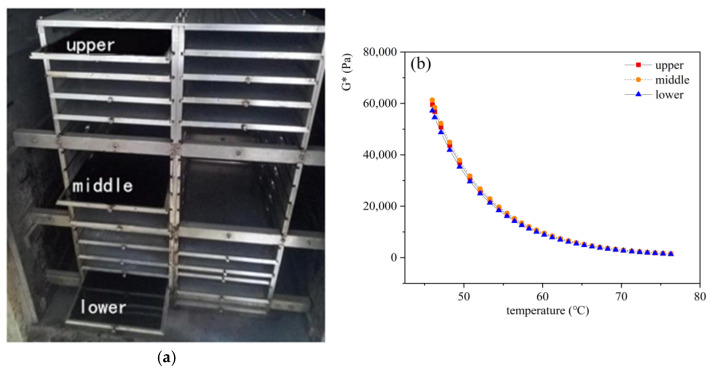
TCAOT asphalt aging test results at different placement locations: (**a**) different placement of asphalt; (**b**) TCAOT asphalt aging test results.

To explore the impact of sample placement on asphalt binder aging effects, this study used three aging trays. Initially, 125 g of ZH-70# asphalt was poured into each tray to create a 1 mm thick asphalt film. These trays were then placed at different levels (upper, middle, and lower) of the aging frame and aged for 5 days at 95 °C to study the rheological properties of aged asphalt binders under different placement conditions, as shown in Figure 10.

The data in the figure demonstrate that under similar test conditions, the G* of asphalt binders at different placement positions were similar, indicating that different placements had no significant impact on asphalt aging effects. It also verified the uniformity of the temperature environment within the aging oven.

A brief analysis of the three parameters (test temperature, sample quantity, sample placement) shows the following: the experimental data show that the complex modulus (G*) of asphalt is significantly changed by increasing the temperature. For example, at 95 °C aging, G* (46 °C) is 1.3 and 2.2 times higher than 85 °C and 70 °C, respectively (see Figure 7). This shows that temperature directly affects the hardening degree and aging depth of asphalt. A high temperature (e.g., 95 °C) results in a short period of time equivalent to long-term aging at low temperatures (e.g., a shortened experiment period), while simply adjusting the sample size or film thickness cannot achieve this nonlinear acceleration. In contrast, sample size and film thickness are tunable parameters in experimental design and are primarily used to optimize operational efficiency rather than to determine the nature of the aging mechanism.

### 3.5. Termination Criteria for Experiments

Linking the test conditions of any laboratory aging simulation method to real-world usage scenarios is crucial. As previously mentioned, RTFOT and PAV correspond to the simulation of short-term and long-term aging stages of asphalt binders, respectively. To achieve complete aging, it is necessary to analyze the correspondence between the TCAOT conditions and the designed service life of asphalt pavements (15 years), i.e., establishing its termination criteria.

This study initially obtained aged asphalt binders from engineering sites, with relevant data from the G6 Jingzang Expressway’s Bailan section. This highway completed its design in July 1999 and opened for traffic in October 2002, reaching its design lifespan. Due to severe defects affecting safety and driving comfort, a major renovation was undertaken in March 2017.

**Figure 11:**
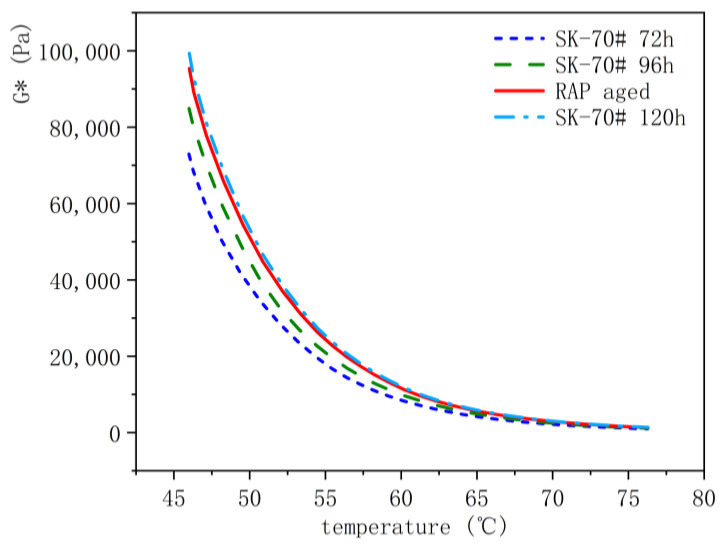
Comparison of rheological properties of field-aged and room-aged asphalt binders (SK-70#).

Subsequently, this study subjected the original SK-70# asphalt to aging using the TCAOT equipment at 95 °C for 72 h, 96 h, and 120 h. The rheological properties of asphalt binders aged in the field and indoors were compared using DSR temperature scanning tests. The results are presented in Figure 11.

From the figure, it is evident that the trend of G* changes in field-aged asphalt binders aligns with laboratory-aged asphalt binders. The field-aged asphalt binder curve closely approximates the 120 h laboratory-aged asphalt binder curve. Thus, the actual aging of asphalt pavements over 15 years corresponds to the aging effect achieved under TCAOT conditions of 95 °C for 120 h. Therefore, the termination criteria for the TCAOT full aging test are 95 °C for 120 h.

### 3.6. Feasibility Analysis of Aging Simulation Equipment

#### 3.6.1. Correlation Analysis with Traditional Methods

To validate TCAOT’s capability to simulate the complete aging process of asphalt binders, this study, in conjunction with traditional RTFOT and PAV methods, investigates their correlation with conventional aging methods.

The experiments used three types of asphalt binders: SK-70#, ZH-70#, and SK-SBS-modified asphalt. Initially, these binders underwent short-term and long-term aging experiments in the TCAOT equipment. The short-term aging experiments involved temperatures of 163 °C (±0.5 °C) for durations of 85 min, 110 min, 120 min, 130 min, and 140 min. Subsequently, long-term aging at 95 °C (±0.5 °C) continued from the completion of short-term aging for durations of 48 h, 72 h, and 96 h. The specific experimental schemes are outlined in Figure 11. DSR temperature scanning tests were conducted to analyze the rheological properties of asphalt binders under different aging devices.

**Figure 12:**
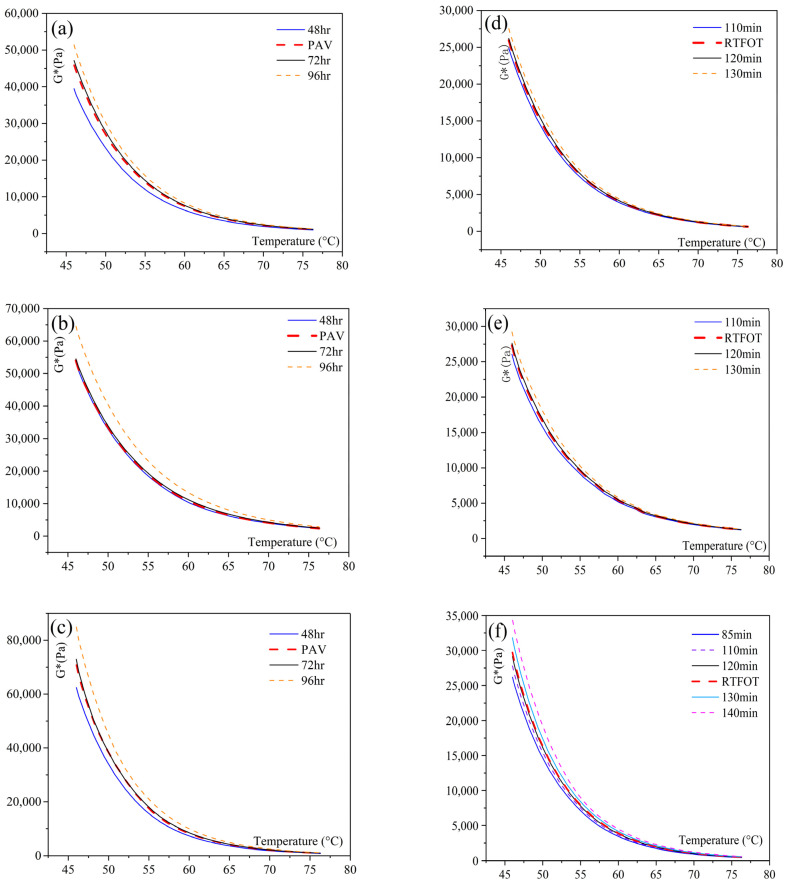
Comparative analysis of aging effect of asphalt binders with different aging equipment: (**a**) SK-70# PAV aging; (**d**) SK-70# RTFOF aging; (**b**) ZH-70# PAV aging; (**e**) ZH-70# RTFOF aging; (**c**) SK-SBS-modified PAV aging; (**f**) SK-SBS-modified RTFOF aging.

Figure 12 illustrates the G* variation curves of different asphalt binders under various aging devices. Comparative analysis of the rheological characteristic curves of the three asphalt types revealed that for short-term aging, the aging effect of TCAOT at 163 °C (±0.5 °C) for 120 min was equivalent to RTFOT. Similarly, for long-term aging, TCAOT’s aging effect matched PAV’s conditions of 95 °C (±0.5 °C) for 72 h.

#### 3.6.2. Necessity Analysis of Complete Aging Simulation

Current research on the aging simulation of asphalt binders usually confines itself to specific aging stages corresponding to the RTFOT and PAV tests. However, simulations for aging levels between RTFOT and PAV or aging that are more severe than PAV are rarely explored. This study aims to analyze the necessity of simulating the complete aging process of asphalt binders using DSR temperature scanning tests.

**Figure 13:**
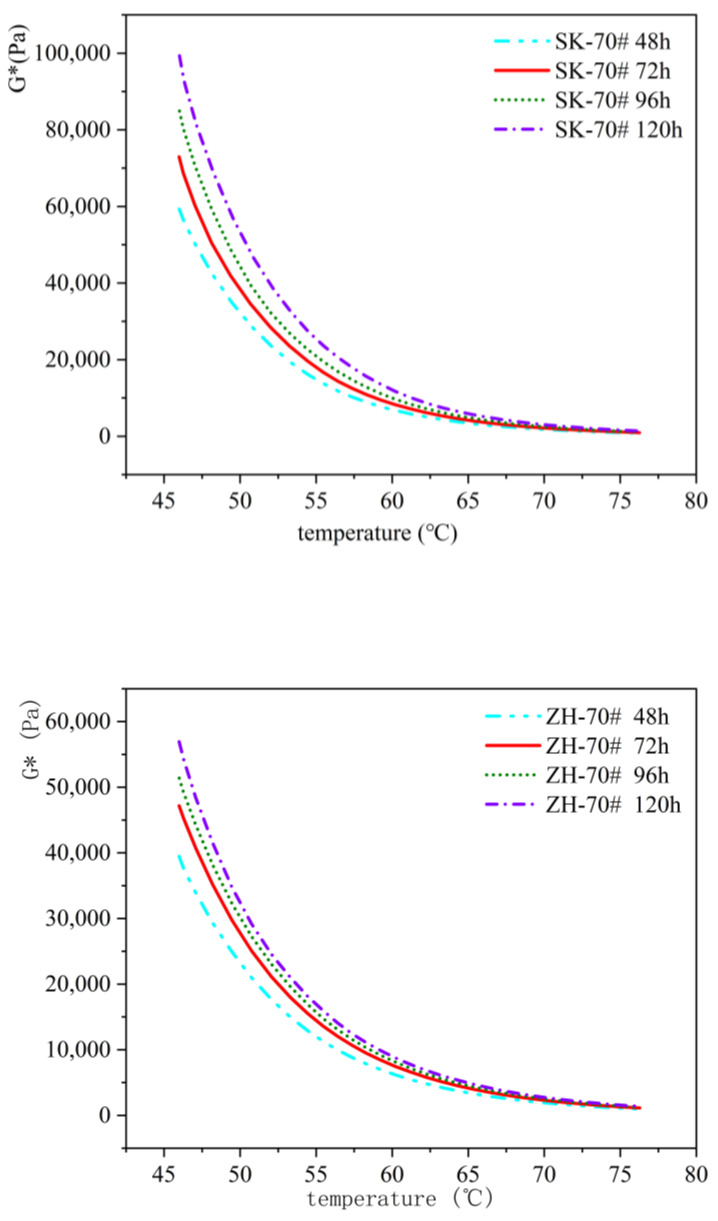
Comparative analysis of complex shear modulus of SK-70# asphalt and ZH-70# asphalt with different aging times.

**Figure 14:**
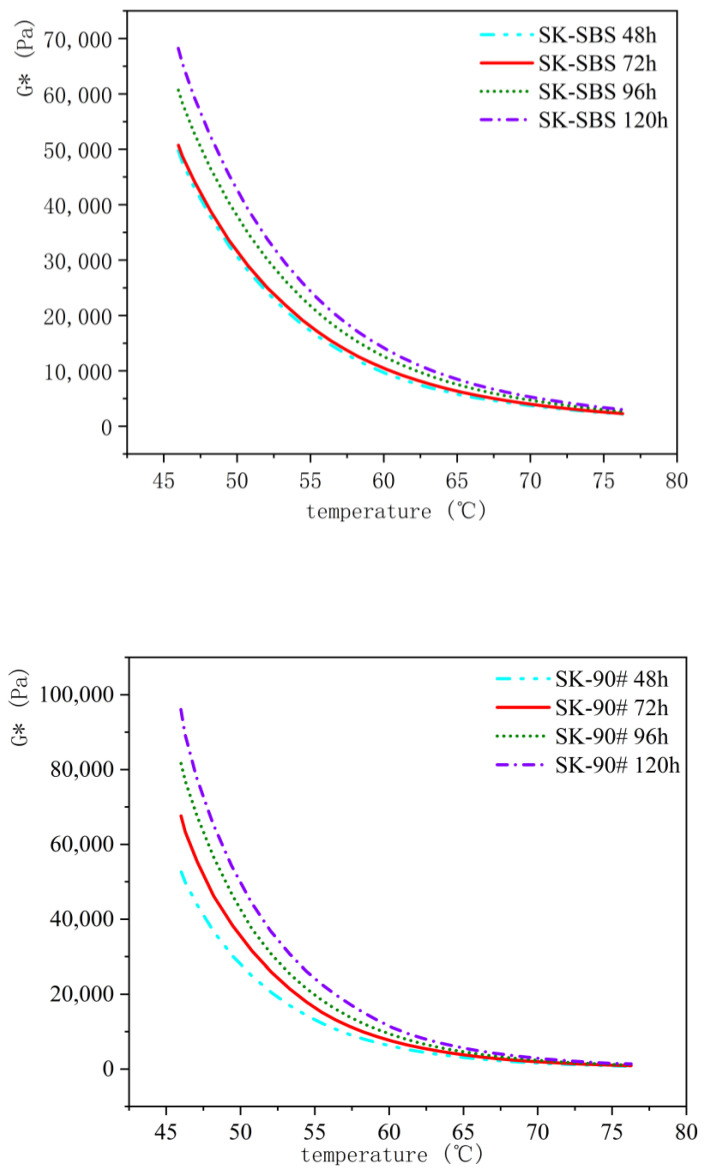
Comparative analysis of complex shear modulus of SK-90# asphalt and SK-SBS-modified asphalt at different aging times.

Four types of asphalt binders—SK-70#, ZH-70#, SK-90#, and SK-SBS modified asphalt—underwent aging tests using TCAOT at 95 °C for durations of 48 h, 72 h (equivalent to PAV aging level), 96 h, and 120 h. The 46 °C complex shear modulus (G*) of asphalt binders at different aging levels was tested, and changes in the rheological properties of asphalt binders at different aging levels were analyzed, as seen in Figure 13 and Figure 14.

The data from the figures show that with an increasing aging duration, the complex shear modulus consistently increases. Referring to the 46 °C complex shear modulus at 48 h as a baseline, the degree of change in G* differed for each asphalt type after 72 h, 96 h, and 120 h of aging: ZH-70# increased by 19%, 30%, and 44%, respectively; SK-70# increased by 23%, 43%, and 67%, respectively; SK-90# increased by 22%, 55%, and 82%, respectively; and SK-SBS modified asphalt increased by 2%, 22%, and 37%, respectively. These findings indicate that beyond 72 h of aging (exceeding the PAV aging level), asphalt binder rheological properties continue to change substantially, with changes exceeding 20%, demonstrating that simulating specific aging stages alone cannot comprehensively reflect the entire aging status of asphalt binders.

Overall, the comprehensive aging simulation of asphalt binders is imperative.

## 4. Conclusions

This study aimed to objectively and accurately simulate the aging process of asphalt binders. Therefore, we developed an aging simulation device, the Temperature-Cycle Aging Oven Test (TCAOT). In-depth research was conducted on key experimental parameters, including the test temperature, sample quantity, sample placement, termination criteria, and feasibility. The major conclusions can be drawn as follows:

(1) Through comparative experiments, the key test parameters for TCAOT were determined as follows: the test condition was thermal oxidation; the test temperature for short-term aging was 163 °C (±0.5 °C); the test temperature for long-term aging was 95 °C (±0.5 °C); the asphalt sample quantity per tray was 125 g (±0.5 g). With 32 aging trays, a total of 4 kg of asphalt could be aged simultaneously. The placement of asphalt samples within the TCAOT had no significant impact on the aging performance. The termination criterion for the full aging test using TCAOT is 95 °C for 120 h.

(2) The complex shear modulus of asphalt binders after only UV aging does not differ much from the original asphalt, whereas the complex shear modulus of asphalt binders after thermal aging is similar to that of aged asphalt with thermal aging and UV aging coupled together.

(3) The actual aging level of asphalt pavements over 15 years corresponds to the aging effect achieved under TCAOT conditions of 95 °C for 120 h.

(4) For short-term aging, the aging effect of TCAOT at 163 °C (±0.5 °C) for 120 min is equivalent to RTFOT. Regarding long-term aging, the aging effect of TCAOT matches PAV under conditions of 95 °C (±0.5 °C) for 72 h.

(5) Using the 46 °C complex shear modulus after 48 h of aging as a reference value, it was observed that, with prolonged aging duration, the rheological properties of aged asphalt continually change.

(6) The types of asphalt selected in this paper were limited, and it is necessary to verify the experimental methods with a variety of asphalt types.

The limitation of this study is that the simulation method does not consider comprehensive environmental factors such as light, traffic load, and water erosion and only focuses on the temperature cycle, which is different from the actual multi-factor coupling aging mechanism experienced by pavements. This also needs to be studied further.

The TCAOT introduced in this article improves experimental efficiency and reduces the number of repetitions through the multi-function integration of a single device, the large-scale production of aging asphalt, and the multi-factor-accelerated aging simulation. TCAOT is easier to clean and costs less to maintain. The road construction industry may be more inclined to adopt such integrated equipment, reducing the dependence on multiple equipment while promoting the development of related technologies and standard updates.

## Figures and Tables

**Table 1:** Technical properties of selected asphalt binders.

Asphalt Types	Abbreviation	Penetration/0.1 mm	Soft Point/°C
Zhonghai 70 matrix asphalt	ZH-70#	74	47.2
SK 70 matrix asphalt	SK-70#	64	47.1
SK-SBS modified asphalt	SK-SBS	59.2	69.4

## Data Availability

The original contributions presented in this study are included in the article. Further inquiries can be directed to the corresponding author.

## Abbreviations

RTFOT	Rotated Thin Film Oven Test
PAV	Pressure Aging Vessel
TCAOT	Temperature-Cycle Aging Oven Test
UV	Ultraviolet
DSR	Dynamic Shear Rheometer

## Nomenclature

G*	Complex shear modulus, Pa
